# Dietary *Codonopsis pilosula* polysaccharides promote gut health and enhance immunity in Hu sheep

**DOI:** 10.3389/frmbi.2025.1589484

**Published:** 2025-06-06

**Authors:** Qing Zhao, Wanning Li, Zilong Liu, Qiao Li, Youji Ma

**Affiliations:** College of Animal Science and Technology, Gansu Agricultural University, Lanzhou, China

**Keywords:** *Codonopsis pilosula* polysaccharides, immunity, gut microbiota, sheep, practical production

## Abstract

In recent years, there has been a growing emphasis on the use of plant polysaccharides in animal husbandry, attracting attention for their distinctive benefits and roles. These natural and eco-friendly feed additives not only enhanced livestock performance but also promoted intestinal health and strengthen immunity. This study utilized 16S rRNA high-throughput sequencing to investigate the effects of dietary *Codonopsis pilosula* polysaccharides on the gut microbiota of Hu sheep. Eighteen 3-month-old Hu sheep with similar body weight (19.60 ± 1.63 kg) and good body condition, were randomly allocated into three groups: a control group (CK) receiving a standard diet, and two trial groups: T1 (supplemented with 0.15% *Codonopsis pilosula* polysaccharides) and T2 (supplemented with 0.3% *Codonopsis pilosula* polysaccharides), with six animals in each group. The pre-trial period lasted for 7 days, followed by an experimental period of 90 days. Results demonstrated that incorporating *Codonopsis pilosula* polysaccharides into the diet markedly increased the acetic acid levels in the ileum. This incorporation was found to enhance the diversity of intestinal flora and influence the species composition and richness of the intestinal microbiota. LEfSe analysis revealed that the genus enriched in the three intestinal segments were primarily *Candidatus_Saccharimonas*, *Christensenellaceae*_R_7_group, *Romboutsia*, and *UCG_005*. The relative expression levels of *Claudin*, *Occludin*, and *ZO-1* mRNA in the T1 group were found to be elevated compared to the CK and T2 groups across all three intestinal segments. In conclusion, these findings indicate that dietary supplementation with *Codonopsis pilosula* polysaccharides not only regulate the intestinal microbial composition of Hu sheep but also enhance their immune capacity by increasing the presence of specific beneficial bacteria, thus fostering the intestinal health of Hu sheep.

## 1 Introduction

In recent years the large-scale development of the breeding industry has led to the widespread use of feed additives (Li et al., 2025; Yu and Li, 2025). However, many of these additives consist of antibiotics, hormones, and other synthetic drugs that, while enhancing the quality of livestock products, pose several issues, including impaired livestock function, reduced immunity, and the development of drug resistance. Additionally, they contribute to environmental pollution (Manyi-Loh et al., 2018) and pose risks to human health (Cheng et al., 2018; Kuppusamy et al., 2018). Therefore, it is been gradually developed to add plant feed ingredients instead of chemical additives, such as flavonoids, polysaccharides and so on (Du et al., 2022; Zhao et al., 2025). In contrast, *Codonopsis pilosula* polysaccharides, primarily derived from Chinese herbs and produced through specialized technology, represent a non-polluting, green alternative. Unlike chemically synthesized drugs, *Codonopsis pilosula* polysaccharides are less likely to induce drug resistance and typically exhibit low toxicity and minimal residue, while preserving their natural structure and biological activity (Alem, 2024). *Codonopsis pilosula* belongs to Chinese herbal plants. These herbal additives not only prevent and treat diseases but also enhance animal performance (Cao et al., 2015). *Codonopsis pilosula*, sourced from nature, can regulate physiological functions and provide multiple benefits, including disease prevention, treatment, immune system enhancement and rich in pharmacological effects (Guo et al., 2024a). As both a therapeutic agent and a green product, *Codonopsis pilosula* is rich in active substances. Its complex and diverse composition contributes to its high efficiency, low toxicity, minimal drug residues, and abundant resource supply (Gao et al., 2018). The primary effects of *Codonopsis pilosula* polysaccharides encompass antimicrobial properties, promotion of growth (Guo et al., 2024b), reduction of inflammation (Chu et al., 2016), enhancement of immune response (Xia et al., 2025), and maintenance of intestinal health (Cao et al., 2022; Zhou et al., 2025). Research indicates that plant-based feed additives have been recognized as effective supplements for non-ruminants (Biswas et al., 2024). This supplementation aids in optimizing and balancing the formulation of livestock and poultry feed, thereby enhancing the nutritional value and utilization efficiency of the feed. The modernization of *Codonopsis pilosula* has led to significant advancements in their application. Numerous studies indicate that these additives enhance growth performance, boost immunity, and improve meat quality, among other benefits. *Codonopsis pilosula* polysaccharides mainly improve the body’s immunity, unlike other bioactive ingredients, such as astragalus polysaccharides, which improves skeletal muscle development and regeneration (Su et al., 2023). Lycium barbarum polysaccharide inhibit lipid oxidation and protein degradation in mutton (Jiangyong et al., 2022), and flavonoids mainly affect the growth of bacteria and play an antibacterial role (Donadio et al., 2021). In the poultry sector, research has demonstrated the positive impacts of *Codonopsis pilosula* polysaccharides on the production performance of laying hens, particularly in enhancing their antioxidant capacity, immunity, and intestinal health (Liu et al., 2021, 2023; Liu and Xie, 2024). Currently, the application of *Codonopsis pilosula* polysaccharides in poultry farming has transitioned from traditional crude usage to a more scientific approach involving precise formulation and standardized production. These natural, eco-friendly feed additives not only effectively enhance the production performance of livestock and poultry but also improve intestinal health and bolster immunity. Considering the global emphasis on green and sustainable development, the integration of *Codonopsis pilosula* polysaccharides in organic farming is expected to emerge as a significant growth area, playing a crucial role in the sustainable advancement of the global livestock industry. Ongoing scientific research and technological innovation are anticipated to expand the applications of *Codonopsis pilosula* in the future, thereby significantly contributing to human health and the sustainable development of animal husbandry.

Gut microorganisms are essential for maintaining the intestinal barrier and play a crucial role in regulating host metabolism (Krishnan et al., 2015), intestinal nutrient digestion, the development and maturation of the immune system (Sanos et al., 2009; Honda and Littman, 2016), and maintaining body homeostasis, thereby significantly contributing to intestinal health (Xiang et al., 2020). Research indicates that *Codonopsis pilosula* polysaccharides can modulate the intestinal flora of lactating piglets, enhancing the population of beneficial intestinal bacteria (Liao, 2024). While numerous studies have investigated the effects of *Codonopsis pilosula* polysaccharides on the rumen of Hu sheep, there is a relative lack of research focusing on their impact on intestinal microorganisms. Therefore, this study aims to analyze the changes and functions of intestinal flora through 16S rRNA sequencing to explore the effects of *Codonopsis pilosula* polysaccharides on the intestinal microbial composition of Hu sheep. The objective is to validate the efficacy of *Codonopsis pilosula* polysaccharides as non-conventional feed options and to provide a theoretical foundation for their practical applications in production.

## 2 Materials and methods

### 2.1 Ethics statement

All animal management and experiments were approved by the Animal Committee of Gansu Agricultural University (GSAU-AEW-2020-0057).

### 2.2 Animals and experimental design

Eighteen 3-month-old Hu sheep, with comparable body weight (19.60 ± 1.63 kg) and in good body condition, were randomly divided into three groups of six sheep each: the CK group (control group, fed a basal diet), the T1 group (trial group I, supplemented with *Codonopsis pilosula* polysaccharides at 0.15% of the basal diet), and the T2 group (trial group II, supplemented with *Codonopsis pilosula* polysaccharides at 0.3% of the basal diet). The pre-test period lasted for seven days, followed by a positive test period of 90 days. Prior to the commencement of the test, the pen was thoroughly sterilized, sheep ear tags were numbered and registered, and the lambs were weighed and assigned to their respective pens. They were fed twice daily at 08:00 and 16:00. The daily *Codonopsis pilosula* polysaccharides were evenly mixed into the concentrate supplement, and water was made available ad libitum. Throughout the feeding phase, all groups were subjected to identical feeding and management protocols.

### 2.3 Preparation of test materials

Extraction of *Codonopsis pilosula* polysaccharides was carried out with reference to the literature (Xu, 2006; Nan et al., 2008). These additives were administered to Hu sheep at rates of 0.15% and 0.3% of the concentrate supplement, respectively. The base ration complied with the nutritional requirements outlined in China’s Meat Sheep Feeding Standard (NY/T 861-2004), with its composition and nutritional levels detailed in **Tables 1**.

**Table 1:** Composition and nutrient levels of basal diet (dry matter basis).

Ingredients	Content/%	Nutrient levels	Content/%
Corn	40.00	ME/(MJ/Kg)^2^	8.57
Wheat bran	9.00	CP	14.51
Peanut seedling	29.00	EE	2.21
Cottonseed meal	5.50	NDF	30.11
Soybean meal	12.50	ADF	19.58
CaHPO4	0.50	Ca	1.01
Vitamin–mineral mix^1^	1.00	P	0.47
Limestone	1.50		
Nacl	1.00		
Total	100.00		

^1^The premix provided the following per kg of diets: Fe (as ferrous sulfate) 75 mg, Cu (as copper sulfate) 14 mg, Zn (as zinc sulfate) 80 mg, Se (as sodium selenite) 0.25 mg, I (as potassium iodide) 0.25 mg, Co 0.30 mg, Mn (as manganese sulfate) 400 mg, VA 3000 IU, VD 500 IU, VE 200 IU.

^2^ME was a calculated value, while the others were measured values.

### 2.4 Sample collection

At the conclusion of the experiment, the test sheep were euthanized. The duodenum, ileum, and cecum were ligated, excised, and rinsed; subsequently, the intestinal contents were scraped, and the tissues were removed using a sterile scalpel. The intestinal tissue was then cleaned with phosphate-buffered saline (PBS) and stored in cryopreservation tubes. These tubes underwent rapid freezing in liquid nitrogen before being immediately placed in liquid nitrogen. Finally, they were transferred to ultra-low-temperature freezers maintained at −80°C for long-term storage.

### 2.5 Determination of ileal VFAs

The concentration of volatile fatty acids (VFA) was determined using gas chromatography, following the method described by Kristensen et al (Kristensen and Harmon, 2004).

### 2.6 16S rRNA sequencing and qRT-PCR

DNA was extracted from the contents of the duodenum, ileum and cecum using a commercial kit. The concentration of DNA was assessed via agarose gel electrophoresis, while DNA quality was evaluated using UV spectrophotometry. Specific primers were employed to purify the PCR products, which were subsequently utilized to generate sequencing libraries. The 16S rRNA sequencing was performed by Biomarker Technologies (Beijing, China).

Total RNA was extracted from the duodenum, ileum, and cecum tissues using Trizol reagent. The concentration and purity of the extracted RNA were assessed with an ultra-micro spectrophotometer. Subsequently, first-strand cDNA was synthesized following the protocol provided with the reverse transcription kit. The mRNA sequences for sheep *ZO-1* and *β-actin* were retrieved from the NCBI database, and primers were designed using Oligo 6.0 software and the NCBI online platform (https://www.ncbi.nlm.nih.gov/). The primers were synthesized by Xi’an Kengke Biotechnology Co., Ltd., with the sequences listed in **Table 2**. The qRT-PCR amplification system comprised a total volume of 20 μL, which included 0.5 μL of each upstream and downstream primer (10 μmol/L), 2× Fast qPCR Master Mixture (Green), and 10 μL of cDNA. The two-step reaction program included an initial denaturation at 95 °C for 2 minutes, followed by 40 cycles of denaturation at 95 °C for 15 seconds and annealing at 60 °C for 30 seconds. The results of the qRT-PCR were analyzed using the 2^−ΔΔCt^ method.

**Table 2:** Primer sequence information.

Genes	Product length	Tm/°C	Primer sequence (5’–3’)
*β-actin*	147 bp	Forward: 58.94	Forward: AGTACCCCATTGAACACGGT
		Revers: 59.04	Revers: CTCTGTTGGCTTTGGGGTTC
*Claudin*	143 bp	Forward: 58.70	Forward: GGTGAAGAAGATGCGGATGG
		Revers: 58.88	Revers: TCTGGTGTTAACGGGTGTGA
*Occludin*	139 bp	Forward: 55.00	Forward: TCCTCATCGTCATCCTGCTC
		Revers: 55.00	Revers: TTCTTCACCCACTCCTCCAC
*ZO-1*	146 bp	Forward: 58.93	Forward: GAAGAGAGCACAGAACGCAG
		Revers: 58.97	Revers: CACTTGTGGCAAGCTGAAGT

### 2.7 Data processing and analysis

The Biomarker Technologies Cloud platform facilitates the processing and analysis of sequencing data, which includes species taxonomic analysis, diversity analysis, significance analysis, and association analysis. We primarily employed Venn diagrams to illustrate the number of unique and shared features across the three intestinal segments. β-diversity analysis was utilized to compare species diversity among samples, focusing on the top 10 colonies at both the phylum and genus levels. Additionally, correlation analysis was conducted to examine the relationship between immune indices and the top 10 colonies at the genus level. The experimental data were analyzed using a one-way ANOVA test with SPSS 27.0 statistical software, and multiple comparisons were performed using Duncan’s method. Results are presented as “mean ± standard deviation” (mean ± SD). Finally, the *P*-value was calculated, with a significance threshold set at *P* < 0.05, indicating that *P* < 0.05 denotes a statistically significant difference, while *P* > 0.05 indicates a lack of significance.

## 3 Results

### 3.1 Variations in volatile fatty acids levels in the ileum of Hu sheep

The concentrations of acetic acid, propionic acid, isobutyric acid, and butyric acid were measured in the ileum of Hu sheep, and the differences in volatile fatty acid content were compared among the groups, as presented in **Table 3**. In the ileum, the concentration of acetic acid was significantly higher in the T1 and T2 groups compared to the CK group (*P* < 0.05). However, the concentrations of propionic acid, isobutyric acid and butyric acid did not exhibit statistically significant differences (*P* > 0.05).

**Table 3:** Differences in the content of volatile fatty acids in the ileum of Hu sheep.

Item	Group ^1^			*P*-value
	CK	T1	T2	
Acetic acid, mmol/L	5.91 ± 2.49^c^	10.86 ± 0.61^b^	15.26 ± 0.18^a^	0.005
Propionic acid, mmol/L	6.64 ± 0.24	4.49 ± 0.02	6.31 ± 0.73	0.057
Isobutyric acid, mmol/L	0.21 ± 0.03	2.99 ± 2.28	1.85 ± 1.29	0.602
Butyric acid, mmol/L	1.13 ± 0.03	0.74 ± 0.37	1.74 ± 0.18	0.102

^1^CK, basal diet; T1, supplemented with *Codonopsis pilosula* polysaccharides according to the basal diet 0.15%; T2, supplemented with *Codonopsis pilosula* polysaccharides according to the basal diet 0.3%.

^a,b,c^Means within a row with different superscript letters are significantly different (*P* < 0.05).

### 3.2 OTU cluster analysis of intestinal microorganisms

**Figure 1:**
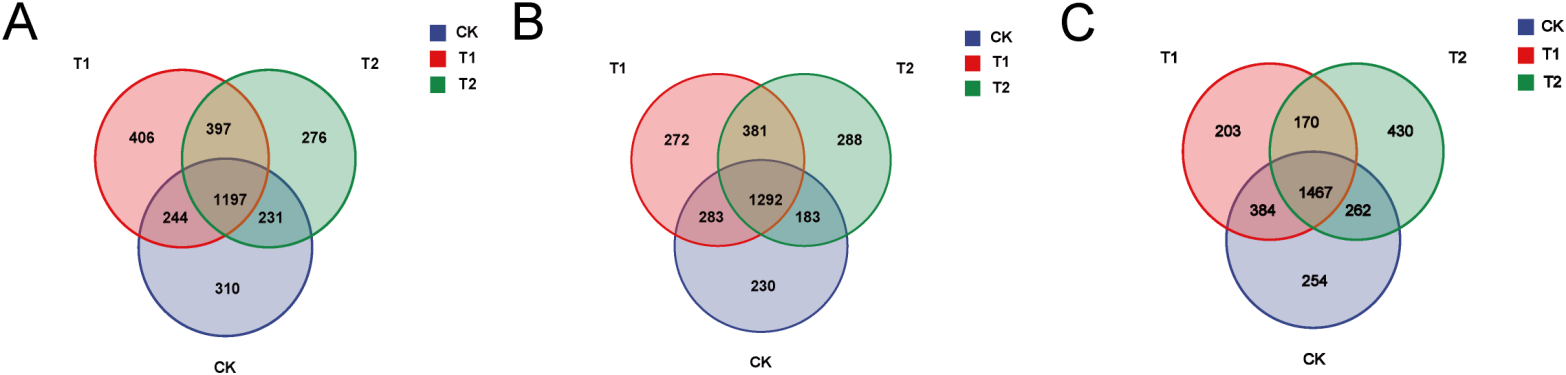
Cluster analysis **(A)** Venn diagram of the three groups in the duodenum. **(B)** Venn diagram of the three groups in the ileum. **(C)** Venn diagram of the three groups in the cecum.

OTU cluster analysis was conducted to quantify the number of operational taxonomic units (OTUs) present in each group. A Venn diagram illustrates the number of OTUs that are common to or unique among the three groups. In the duodenal samples, the unique OTUs for the CK group, T1 group, and T2 group were 310, 406, and 276, respectively (**Figure 1A**). For the ileum samples, the unique OTUs for the CK group, T1 group, and T2 group were 230, 272, and 288, respectively (**Figure 1B**). In the cecum samples, the unique OTUs for the CK group, T1 group, and T2 group were 254, 203, and 430, respectively (**Figure 1C**). These results indicate that dietary supplementation with *Codonopsis pilosula* polysaccharides can significantly influence the gut microbiota of Hu sheep.

### 3.3 Analysis of the alpha diversity of intestinal microorganisms

As shown in **Table 4**, in the duodenum, the differences in the ACE, Chao1, and Simpson indices between the T1 and T2 groups were not significant compared to the CK group (*P* > 0.05). However, the differences in the Shannon index were significant (*P* < 0.05). In the ileum, no significant differences were observed among the groups for the Shannon, Simpson, ACE and Chao1 indices (*P* > 0.05). In the cecum, when compared to the CK group, the differences in the ACE, Shannon, and Simpson indices between the T1 and T2 groups were not significant (*P* > 0.05), whereas the differences in the Chao1 index were significant (*P* < 0.05).

**Table 4:** Effects of *Codonopsis pilosula* polysaccharides on α-diversity index of three intestinal segments of Hu sheep.

Item	Diversity index	Group ^1^			*P*-value
		CK	T1	T2	
Duodenum	ACE	1273.03 ± 113.89	1296.54 ± 98.15	1150.39 ± 58.32	0.11
	Chao1	1143.85 ± 162.27	1271.76 ± 189.88	1169.28 ± 67.81	0.52
	Simpson	0.97 ± 0.25	0.97 ± 0.17	0.98 ± 0.01	0.24
	Shannon	5.80 ± 0.44^b^	7.27 ± 0.96^a^	7.76 ± 0.45^a^	0.01
Ileum	ACE	1166.55 ± 162.81	1233.93 ± 468.37	1319.82 ± 75.82	0.76
	Chao1	1177.59 ± 180.71	1240.25 ± 462.87	1208.89 ± 205.21	0.97
	Simpson	0.97 ± 0.02	0.99 ± 0.01	0.97 ± 0.03	0.43
	Shannon	7.14 ± 0.74	8.08 ± 1.02	7.05 ± 1.17	0.35
Cecum	ACE	1471.12 ± 111.31	1414.73 ± 88.55	1345.72 ± 81.05	0.30
	Chao1	1494.87 ± 110.79^a^	1445.04 ± 83.46^a^	1243.93 ± 93.80^b^	0.02
	Simpson	0.98 ± 0.02	0.98 ± 0.01	0.97 ± 0.03	0.30
	Shannon	8.09 ± 0.62	8.14 ± 0.12	7.98 ± 0.08	0.90

^1^CK, basal diet; T1, supplemented with *Codonopsis pilosula* polysaccharides according to the basal diet 0.15%; T2, supplemented with *Codonopsis pilosula* polysaccharides according to the basal diet 0.3%.

^a,b^Means within a row with different superscript letters are significantly different (*P* < 0.05).

### 3.4 Analysis of the β-diversity of intestinal microorganisms

**Figure 2:**
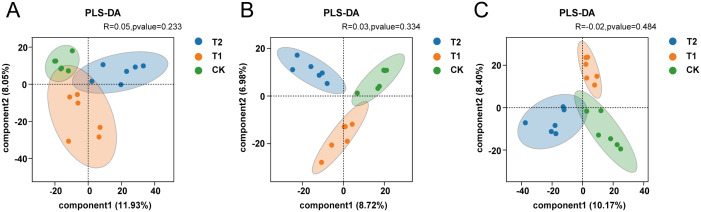
Analysis of diversity **(A)** β-diversity analysis of the three groups in the duodenum. **(B)** β-diversity analysis of the three groups in the ileum. **(C)** β-diversity analysis of the three groups in the cecum.

The analysis of β-diversity in intestinal microorganisms was performed using Partial Least Squares Discriminant Analysis (PLS-DA), which is based on the classical partial least squares regression model. This analysis effectively highlights the differences in microbial community structures among samples from various groups. In the three intestinal segments examined, the distribution of intestinal microorganisms among the CK, T1, and T2 groups exhibited a clear separation between the upper and lower parts segments of the groups, with significant aggregation observed within each group (*P* < 0.05). These findings suggest that the microbial community structures of the three groups are distinct, and that the incorporation of *Codonopsis pilosula* polysaccharides into the diet may influence the composition of gut microorganisms in Hu sheep (**Table 4**; **Figure 2**).

### 3.5 Effect of *Codonopsis pilosula* polysaccharides on microbial phylum levels in three intestinal segment of Hu sheep

**Figure 3:**
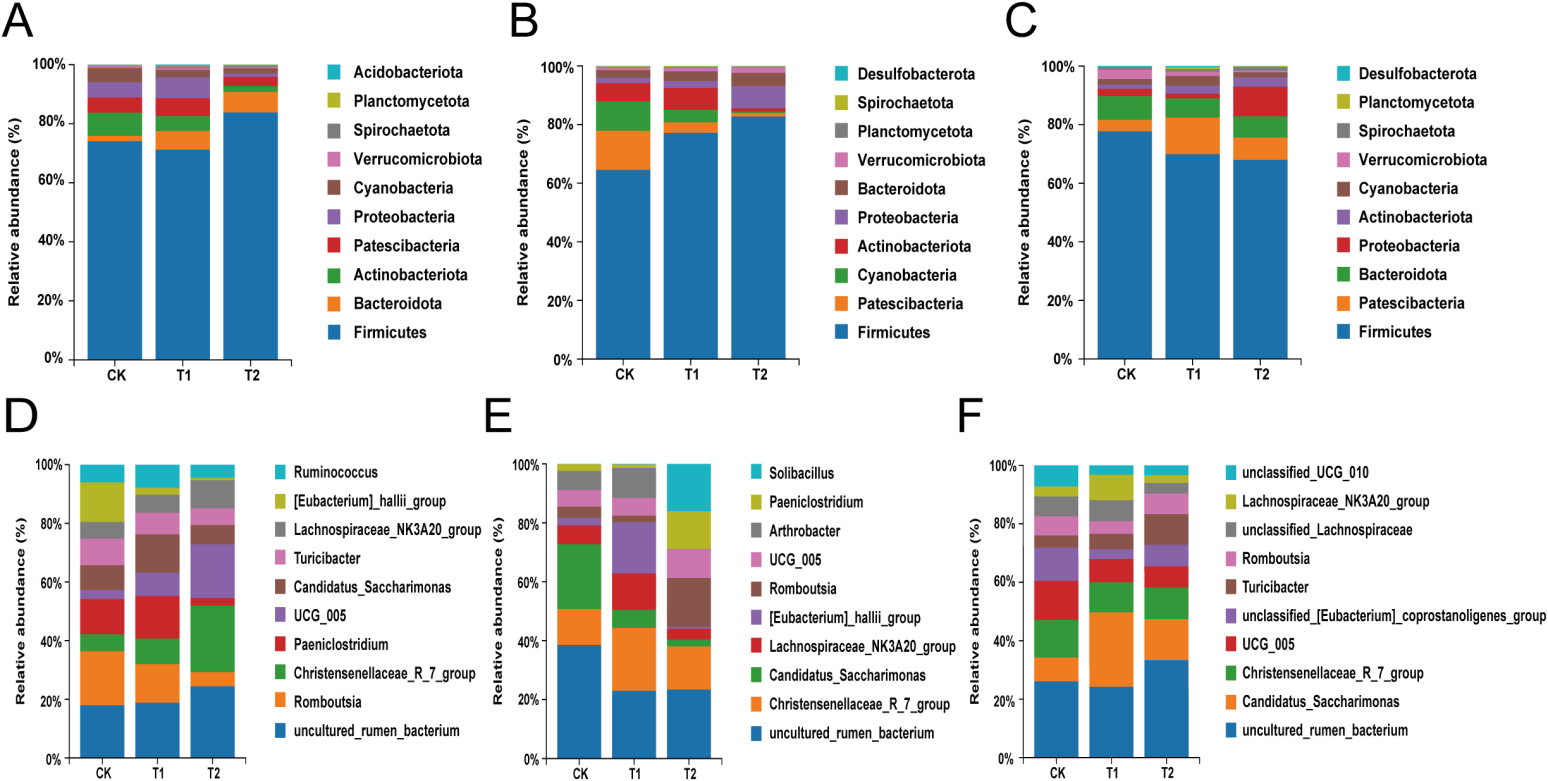
Analysis of differences in species composition of intestinal flora. **(A)** Composition of the duodenum at the phylum level. **(B)** Composition of the ileum at the phylum level. **(C)** Composition of the cecum at the phylum level. **(D)** Composition of the duodenum at the genus level. **(E)** Composition of the ileum at the genus level. **(F)** Composition of the cecum at the genus level.

The top ten species in terms of total abundance were analyzed in depth at the phylum level through species annotation and statistical counting of valid sequences obtained at various taxonomic levels. Firmicutes were identified as the dominant bacteria across all groups within the duodenum of Hu sheep. The relative abundance of Bacteroidota exhibited a gradual increase with higher levels of additives (**Figure 3A**). In the ileum of Hu sheep, Firmicutes continued to dominate across all groups, with a similar trend observed for Bacteroidota, which increased in relative abundance alongside the amount of additive administered (**Figure 3B**). In the cecum, the predominant phyla among all groups of Hu sheep included Firmicutes, Patescibacteria, Bacteroidota, and Actinobacteriota (**Figure 3C**). The genus with the highest relative abundance in the duodenum of the CK group was *Romboutsia*, while *uncultured_rumen_bacterium* was the most abundant in the T1 and T2 groups. The relative abundance of *Christensenellaceae*_R_7_group and *UCG-005* progressively increased with higher additive levels (**Figure 3D**). At the ileal microbial genus level, *uncultured_rumen_bacterium* and *Christensenellaceae*_R_7_group emerged as the dominant genera, followed by *[Eubacterium]_hallii_group* and *Lachnospiraceae_NK3A20_group*. Notably, the genera *Romboutsia*, *Paeniclostridium* and *Solibacillus* were significantly elevated (*P*<0.05) in the T2 group (**Figure 3E**). In the cecum, the dominant genus in the CK group included *uncultured_rumen_bacterium*, *Christensenellaceae*_R_7_group, and *UCG_005*, whereas in the T1 and T2 groups, the dominant genera consisted of *uncultured_rumen_bacterium*, *Candidatus_Saccharimonas* and *Christensenellaceae*_R_7_group (**Figure 3F**).

### 3.6 LEfSe analysis

**Figure 4:**
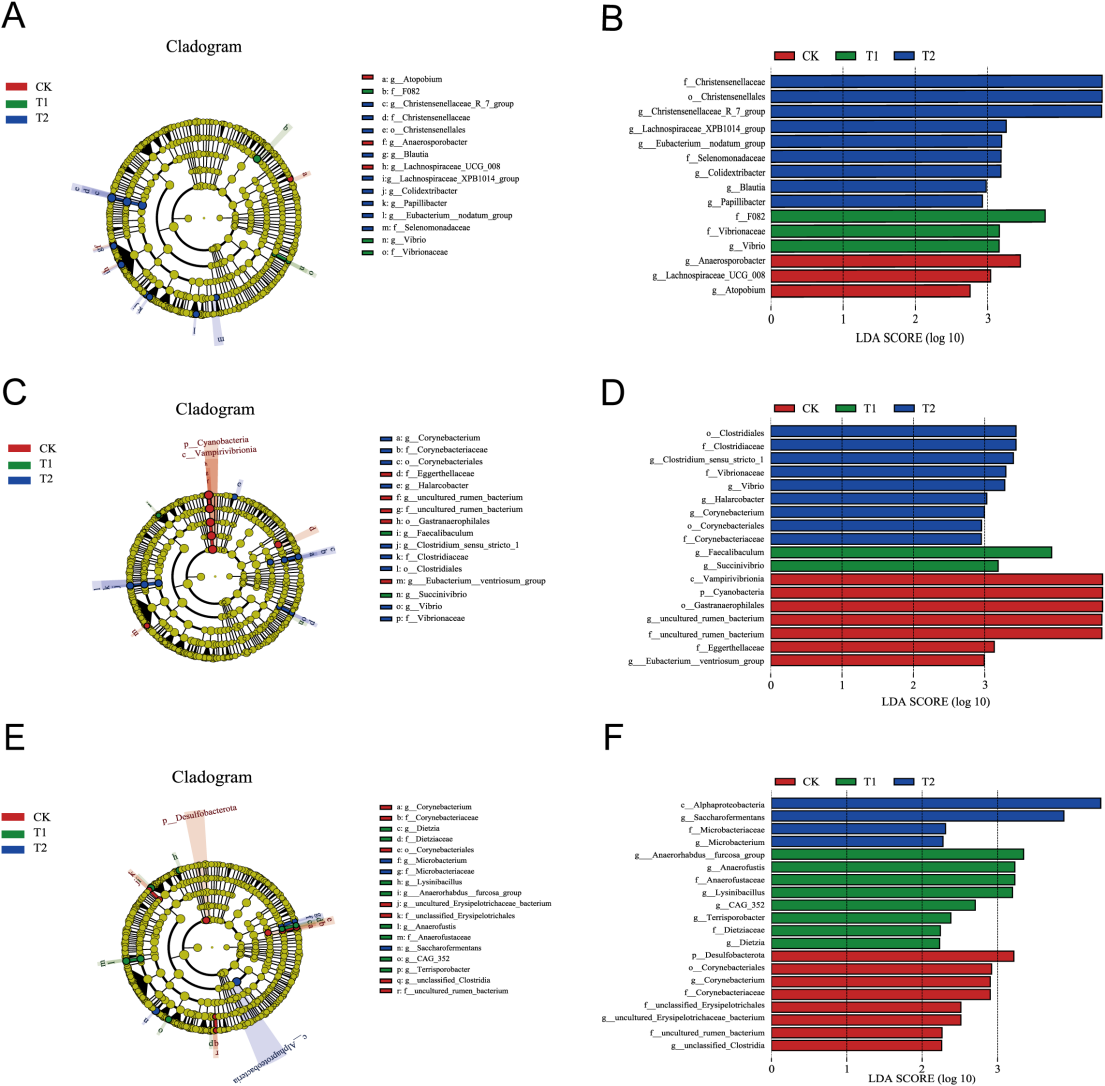
Significance analysis of differences between the three groups. **(A)** LEfSe analysis of duodenum. **(B)** LDA score chart of the duodenum. **(C)** LEfSe analysis of ileum. **(D)** LDA score chart of the ileum. **(E)** LEfSe analysis of cecum. **(F)** LDA score chart of the cecum.

Significantly altered gut microorganisms were identified through LEfSe analysis, focusing on those with an LDA score greater than 2 (LDA > 2) at the genus level. In the duodenum, three, one, and six groups were enriched in the CK, T1, and T2 groups, respectively, with the highest LDA score impacts attributed to *g_Anaerosporobacter*, *g_Vibrio*, and *g_Christensenellaceae*_R_7_group (**Figures 4A, B**). In the ileum, two, two, and four groups were enriched in the CK, T1, and T2 groups, respectively, with the highest LDA score impacts associated with *g_uncultured_rumen_bacterium*, *g_Faecalibaculum*, and *g_Clostridium_sensu_stricto_1* (**Figures 4C, D**). In the cecum, three, six, and two groups were enriched in the CK, T1, and T2 groups, respectively, with the highest LDA score impacts linked to *g_Corynebacterium*, *g_Anaerorhabdus_furcosa_group*, and *g_Saccharofermentans* (**Figures 4E, F**).

### 3.7 Effects on the intestinal mucosal barrier of intestinal tissues in Hu sheep

**Figure 5:**
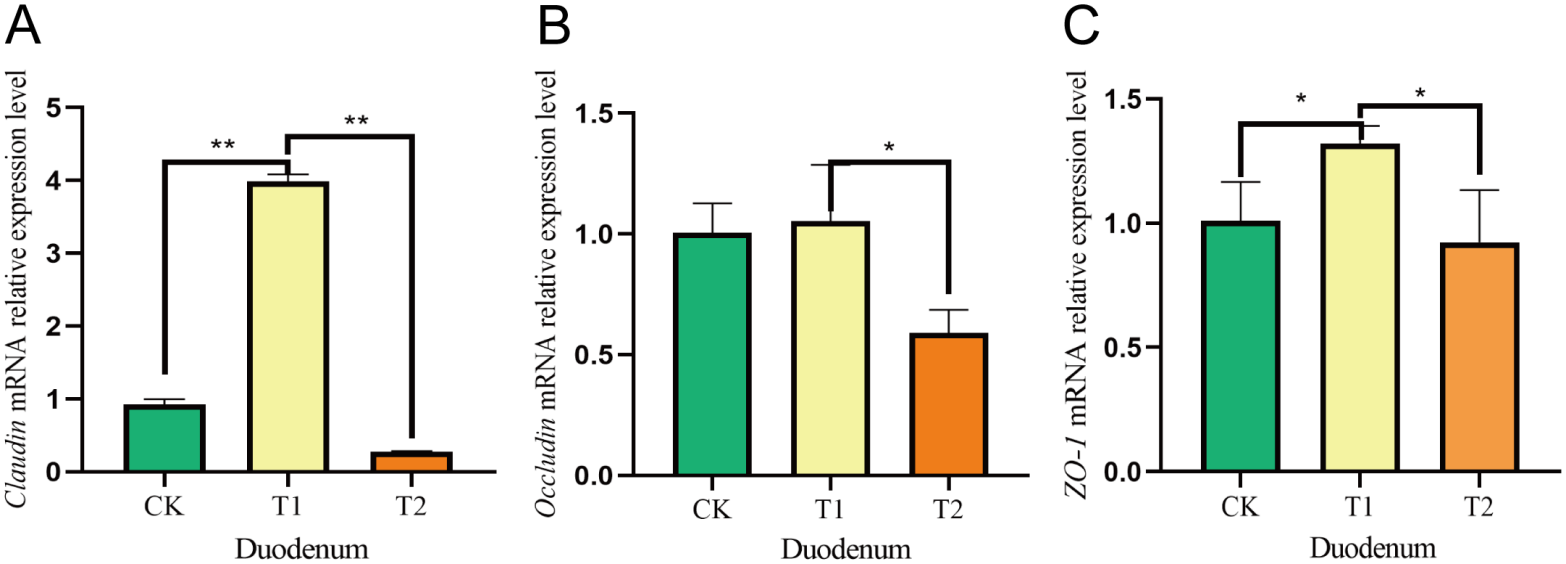
Effects of adding *Codonopsis pilosula* polysaccharides to diets on the expression of intestinal barrier related genes in Hu sheep. **(A)** Effect of *Claudin* mRNA expression in duodenum. **(B)** Effect of *Occludin* mRNA expression in duodenum. **(C)** Effect of *ZO-1* mRNA expression in duodenum. * represents significant difference ;** represents extremely significant difference.

**Figure 6:**
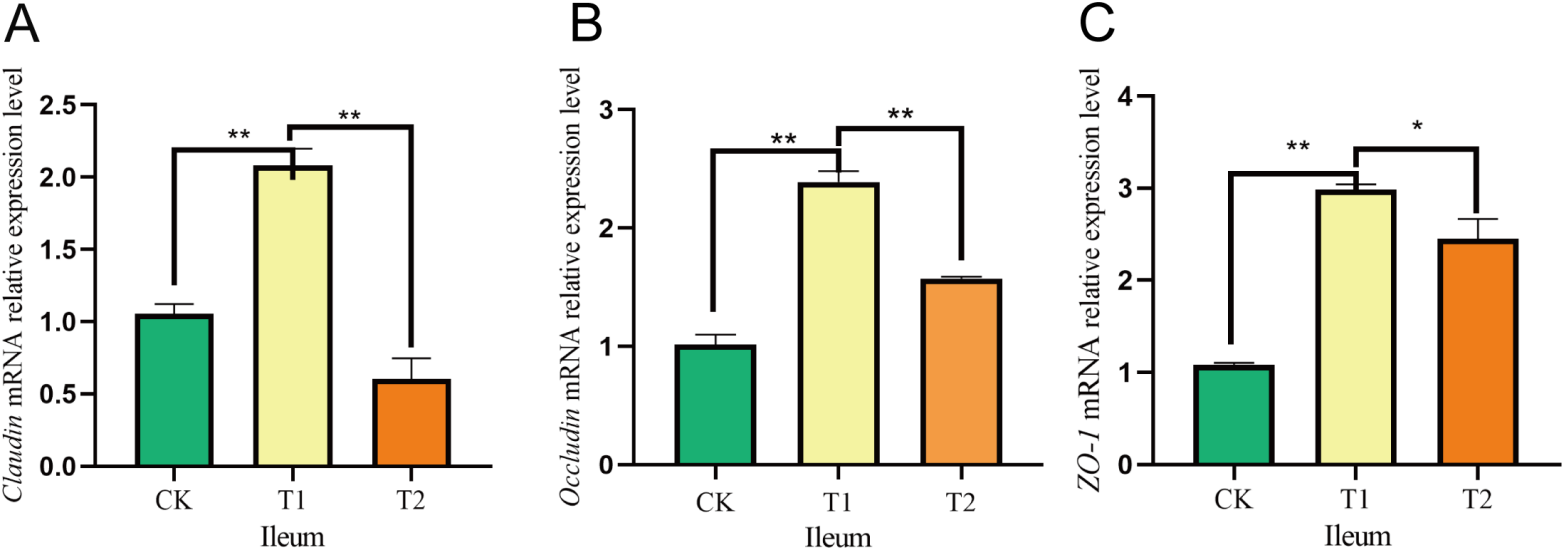
Effects of adding *Codonopsis pilosula* polysaccharides to diets on the expression of intestinal barrier related genes in Hu sheep. **(A)** Effect of *Claudin* mRNA expression in ileum. **(B)** Effect of *Occludin* mRNA expression in ileum. **(C)** Effect of *ZO-1* mRNA expression in ileum. * represents significant difference ;** represents extremely significant difference.

In the duodenum, the *Claudin* mRNA expression levels were considerably increased in the T1 group compared to both the CK and T2 groups. Additionally, the mRNA expression of *Occludin* showed a significant rise in the T1 group relative to the T2 group, although no notable difference was noted in comparison to the CK group. Furthermore, the relative expression of *ZO-1* mRNA was significantly greater in the T1 group than in both the CK and T2 groups (**Figure 5**). In the ileum, both *Claudin* and *Occludin* mRNA levels were notably elevated in the T1 group in comparison to the CK and T2 groups. Moreover, the *ZO-1* mRNA expression was significantly greater in the T1 group when assessed against both the CK and T2 groups (**Figure 6**).

## 4 Discussion

In ruminants, volatile fatty acids are primarily produced in the rumen and intestine (Svartström et al., 2017). The duodenum is vital for absorbing digestive products from sugars, fats, and proteins, while the production and absorption of volatile fatty acids mainly occur in the posterior part of the intestine (Song et al., 2021). In our study, we measured the volatile fatty acid content in the ileum to investigate the effects of *Codonopsis pilosula* polysaccharides on the intestinal tract. The results demonstrated that the acetic acid content was significantly higher in the T1 and T2 groups compared to the CK group. Acetic acid, a short-chain fatty acid (SCFA), is produced by the bacterial fermentation of indigestible carbohydrates and influences lipid, carbohydrate and protein metabolism (Frampton et al., 2020). SCFAs are essential for maintaining intestinal microflora and are necessary substances for balancing the intestinal internal environment (Van Hoek and Merks, 2012). Prior research has clearly shown that SCFAs could significantly contribute to the prevention and management of intestinal disorders (Blouin et al., 2011). Therefore, we hypothesized that the addition of *Codonopsis pilosula* polysaccharides to the ration could enhance the digestive capacity of the intestinal tract in Hu sheep.

Alpha diversity is primarily employed to assess species richness and evenness within a specific region, utilizing metrics such as the Chao1, ACE, Shannon, and Simpson indexes. Elevated values of the Chao1 and ACE indexes indicate greater species richness, whereas higher values of the Shannon index reflect increased community diversity. In contrast, lower values of the Simpson index suggest a higher level of community diversity (Hughes et al., 2001; Kim et al., 2017). It has been established that the diversity of the gut microbial community is essential for the health of the host organism and plays a significant role in immunization (Yadav and Jha, 2019). In this study, we observed that the α-diversity Shannon index of the duodenum and the α-diversity Chao1 index of the cecum were significantly higher (*P* < 0.05) in Hu sheep that received *Codonopsis pilosula* polysaccharides compared to the CK group. The β-diversity analysis revealed a clear separation of microorganisms between groups, accompanied by aggregation within groups. This finding indicates that the inclusion of *Codonopsis pilosula* polysaccharides in the diet may alter the intestinal microbial composition of Hu sheep and enhance the diversity of microbial communities in the duodenum and cecum, thereby fostering the development of immunity-related microbial communities in the intestinal tract and ultimately improving the immune capacity of the Hu sheep.

Significant difference analysis at the phylum level revealed that Firmicutes was the predominant flora across all three intestinal segments. This finding aligns with the observations of Zhong et al. (2022), who identified Firmicutes as the primary dominant flora in the intestinal tract of sheep. Additionally, Zhang et al. (2018) utilized high-throughput sequencing technology to demonstrate that Firmicutes was the dominant flora in the cecum of small-tailed cold sheep. Members of Firmicutes actively contribute to the host immune system by stimulating immune responses and influencing the activity of immune cells (Ross et al., 2024). They efficiently break down polysaccharides by secreting various enzymes (Gharechahi et al., 2023), degrading fibers and cellulose (Thoetkiattikul et al., 2013) and fermenting polysaccharides to produce short-chain fatty acids(SCFAs). These SCFAs are crucial for maintaining normal intestinal function, supporting intestinal health, regulating host metabolism, and facilitating nutrient digestion and absorption (Wang et al., 2019; Hu et al., 2023). The results also indicated that the relative abundance of Bacteroidetes were higher in each group. Bacteriophages are beneficial bacteria capable of metabolizing polysaccharides and oligosaccharides, thereby providing nutrients to the host (Zafar and Saier, 2021). The incorporation of LBP into the diets of weaned piglet, as a substitute for antibiotics, has been demonstrated to enhance antioxidant capacity, bolster immunity, and modulate the gut microbial composition of these animals (Yin et al., 2021). Furthermore, Zhang et al. (2024) found that the addition of shiitake mushroom polysaccharides to milk replacer powder elevated serum levels of IgA, IgG, and IgM in calves, thereby improving their immune function. In the present study, as the content of *Codonopsis pilosula* polysaccharides gradually increased, was associated with a gradual increase in the relative abundance of Firmicutes in the ileum, corresponding to the increased quantity of *Codonopsis pilosula* polysaccharides. This finding suggests that *Codonopsis pilosula* polysaccharides have influenced the relative abundance of Firmicutes, subsequently impacting intestinal fermentation. Such alterations have not only enhanced the digestion and absorption of rations in Hu sheep but also improved their immune response.

In this experiment, the analysis of significant differences at the genus level revealed that the dominant genera in the three intestinal segments were *Romboutsia*, *UCG_005*, and *Christensenellaceae*_R_7_group. This finding aligns with the results reported by Ma et al (Ma et al., 2022), which indicated that the dominant genera in the hindgut (ileum and cecum) of sheep are *UCG_005* and *Christensenellaceae*_R_7_group. By LEfSe analysis, in the ileum, *g_Faecalibaculum* and *g_Clostridium_sensu_stricto_1* were significantly enriched in the T1 and T2 groups compared to the CK group. *g_Faecalibaculum* possesses anti-inflammatory properties and is a key producer of butyric acid, which plays a crucial protects the digestive system and maintains intestinal health (Sokol et al., 2008). *g _Clostridium_sensu_stricto_1* is an anaerobic bacterium that has been associated with the integrity of the intestinal barrier (Kasahara et al., 2018). Additionally, in the cecum, both *g_Anaerorhabdus_furcosa_group* and *g_Saccharofermentans* were significantly enriched in T1 and T2 groups compared to the CK group. The *g_Anaerorhabdus_furcosa_group*, which belongs to the phylum Bacteroidota, is known for producing substantial amounts of acetic acid, thereby promoting intestinal health. *g_Saccharofermentans*, a member of the phylum Firmicutes, plays a role in enhancing gut microbiota diversity. *Romboutsia* is primarily involved in regulating of lipid metabolism, as well as fat digestion and absorption, and is classified as a probiotic bacterium. In our study, the relative abundance of *Romboutsia* in the ileum and cecum was significantly higher in the T2 group compared to the CK group, suggesting that *Codonopsis pilosula* polysaccharides enhanced the population of beneficial intestinal bacteria in Hu sheep. *UCG-005*, which belongs to the family Ruminalococcaceae, includes members that play a critical role in the degradation and digestion of cellulose and starch in animals (Chassard et al., 2012; Cann et al., 2016; Huang et al., 2022). Consequently, the family Ruminalococcaceae is regarded as potentially beneficial bacteria capable of regulating the gut’s internal environment and may be linked to immune modulation (Cheng et al., 2023). The genus *Christensenellaceae*_R_7_group part of the family Christensenellaceae, is often recognized for its positive effects on maintaining intestinal health and plays an important role in the degradation of cellulose and hemicellulose (Xin et al., 2019). Research has shown that *Christensenellaceae*_R_7_group is associated with feed efficiency and may participate in host metabolic processes, with significant implications for gut health and host energy balance (Pang et al., 2022). The Christensenellaceae family can use a variety of sugars, the most important fermentation end products are acetic acid and butyric acid (Morotomi et al., 2012). The analysis of differences at the genus level revealed a gradual increase in the relative abundance of *UCG-005* and *Christensenellaceae*_R_7_group in both the duodenum and ileum. This finding suggests that the incorporation of *Codonopsis pilosula* polysaccharides into the diet enhances the relative abundance of beneficial gut bacteria, thereby influencing gut flora composition, increasing gut flora biodiversity, and supporting intestinal health.

In ruminants, gut microbes significantly influence gut barrier function by regulating host–microbe relationships (Lyte et al., 2018). The tight junction proteins *Occludin*, *Claudin* and *ZO-1* family are critical components of the barrier (Zhang et al., 2022; Li et al., 2024). Given that the small intestine serves as the primary digestive organ alongside the tumor, and later in our researches we found that the major phylum and genus level microorganisms were immunologically relevant, we investigated the relative gene expression of *Occludin*, *Claudin* and *ZO-1* following the incorporation of *Codonopsis pilosula* polysaccharides into the diet. Our results indicated that the relative expression levels of *Occludin*, *Claudin* and *ZO-1* in the duodenum and ileum were higher in the T1 group compared to the CK and T2 groups. Research indicates that feeding mice an appropriate amount of *Codonopsis pilosula* polysaccharides can boost T lymphocytes and beneficially regulate intestinal mucosal immunity. Conversely, high doses may inhibit these cells (Wang et al., 2024). This is similar to our research findings, where the gene expression in the T1 group was higher than that in the T2 group, possibly because high doses of *Codonopsis pilosula* polysaccharides may cause overactivation of the immune system, leading to abnormal immune responses and gastrointestinal dysfunction. We hypothesize that the inclusion of *Codonopsis pilosula* polysaccharides in the diet may enhance the intestinal barrier, however, the specific mechanisms underlying this effect require further investigation.

## 5 Conclusions

The results of this study indicated that *Codonopsis pilosula* polysaccharides significantly increase the levels of acetic acid in the ileum. Additionally, *Codonopsis pilosula* polysaccharides influence the diversity of intestinal microorganisms in the duodenum and cecum of Hu sheep. At the phylum level, Firmicutes were the dominant bacteria across all groups in the three intestinal segments. At the genus level, the predominant genera across the three intestinal segments included *Romboutsia*, *UCG-005*, and *Christensenellaceae*_R_7_group. Ultimately, this study concluded that the addition of *Codonopsis pilosula* polysaccharides to the diet might modulate the intestinal microbial composition of Hu sheep, improve intestinal barrier function, and enhance their immune competence by increasing the abundance of specific dominant flora. Therefore, the appropriate amount of *Codonopsis pilosula* polysaccharides can be used as a green feed additive, which is helpful to promote the development of animal husbandry to a more ecological, environmental protection and sustainable direction, in line with the modern green agricultural concept.

## Data Availability

The original contributions presented in the study are publicly available. This data can be found here: NCBI, accession PRJNA1202939.
